# Factors Influencing a Favorable Outcome for RFA of Huge Benign Thyroid Nodules: Preliminary Results and Short-Term Evaluation

**DOI:** 10.1155/2023/9021903

**Published:** 2023-12-14

**Authors:** Chun-Hua Chiu, Sheng-Dean Luo, Pi-Ling Chiang, An-Ni Lin, Cheng-Kang Wang, Chen-Kai Chou, Shun-Yu Chi, Meng-Hsiang Chen, Wei-Che Lin

**Affiliations:** ^1^Department of Diagnostic Radiology, Kaohsiung Chang Gung Memorial Hospital and Chang Gung University College of Medicine, Kaohsiung, Taiwan; ^2^Department of Otolaryngology, Kaohsiung Chang Gung Memorial Hospital and Chang Gung University College of Medicine, Kaohsiung, Taiwan; ^3^Division of Endocrinology and Metabolism, Department of Internal Medicine, Kaohsiung Chang Gung Memorial Hospital and Chang Gung University College of Medicine, Kaohsiung, Taiwan; ^4^Department of Surgery, Kaohsiung Chang Gung Memorial Hospital and Chang Gung University College of Medicine, Kaohsiung, Taiwan; ^5^Department of Radiology, Jen-Ai Hospital, Taichung, Taiwan

## Abstract

**Objective:**

This study aimed to investigate potentially favorable factors influencing the therapeutic success of radiofrequency ablation (RFA) of huge benign thyroid nodules (BTNs) (volume >100 ml) and to evaluate the feasibility of RFA as an alternative treatment modality for patients unable or unwilling to undergo surgery.

**Methods:**

This retrospective study evaluated a total of 868 patients, of which 22 patients had huge BTNs who underwent ultrasound-guided moving shot RFA treatment between May 2017 and January 2022. The huge BTNs were categorized into two groups according to a post-RFA treatment volume reduction ratio (VRR) of >80% and <80% at 6 months. Factors influencing these huge BTNs were reviewed, analyzed, and correlated with treatment effectiveness between the two groups.

**Results:**

The factors influencing an effective VRR included huge BTNs located on the left side (OR 7.875, *p* = 0.03), predominant solid/spongiform nodules (OR 7.875, *p* = 0.03), and higher initial ablation rate (IAR) (*p* = 0.028). Multivariable logistic regression revealed predominant solid/spongiform nodule and the higher IAR were associated with the advanced VRR.

**Conclusion:**

RFA was effective at decreasing the volume of huge BTNs with an acceptable complication rate. The BTN characteristics correlated with a better VRR at the 6-month short-term follow-up were predominant solid/spongiform BTNs and those with the first time ablation treatment initial ablation rate. Nevertheless, regarding the higher regrowth rate of these groups of patients who may need to be treated more times, RFA can only be a feasible alternative treatment modality for patients unable or unwilling to undergo operation.

## 1. Introduction

Thyroid nodular disease is a common endocrine disorder, the discovery and treatment of which have become more successful due to the application of neck ultrasound scanning in clinical practice. As thyroid surgery is associated with various general anesthesia risks, operation scar, and hypoparathyroidism, minimally invasive image-guided ablation has been suggested as a viable alternative treatment option [1]. In recent years, a growing number of studies have investigated the treatment of benign thyroid nodules (BTNs) with minimally invasive image-guided thermal ablation, including radiofrequency ablation (RFA), microwave ablation, laser ablation (LA), and high-intensity-focus ultrasound (HIFU) [1]. According to several guidelines and consensus statements, RFA may be used as a first-line treatment or as an alternative to surgery for patients with solid nonfunctioning thyroid nodules [2–11]. Furthermore, RFA treatment of BTNs can achieve a significant volume reduction ratio (VRR) with symptom and cosmetic improvements observed between 1 and 6 months, with a notably low recurrence rate at 2 years [12–14]. Compared to surgical resection, the relatively low complication rate and minimal scar [13, 15] have made RFA a viable treatment modality for general-sized BTNs in patients unable or unwilling to undergo surgery [16].

Although no definitive criteria regarding nodule size or volume have been established for thyroid RFA treatment [3], it has been reported that patients presenting with BTNs exceeding 2 cm in diameter suffer from a variety of symptoms, clinical concerns, and cosmetic issues [17]. Meanwhile, relevant literature addressing the treatment of huge BTNs with RFA is lacking. A number of previous studies have defined large BTNs as presenting with a volume >30 ml [18–23], the largest of which may reach a volume of 104.5 ml [23]. In light of this, we here define BTNs with a volume >100 ml as huge BTNs. Both surgery and RFA are effective methods for treating nodule-related clinical issues. Surgery remains the standard treatment for patients with symptomatic large BTNs as surgery can completely remedy the compressive symptoms and harvest the whole huge BTN specimen to make sure the final pathology is benign. In contrast, RFA does not have the effect to resolve the BTNs' symptoms entirely [15, 24–26]. Nonetheless, the elevated risks associated with anesthesia may be unacceptable for patients presenting with certain comorbidities. RFA is thus a common alternative treatment for moderately sized BTNs [25, 27]. However, studies have yet to investigate the factors affecting the successful RFA treatment of large BTNs. Although several previous studies have indeed investigated large BTNs, the relatively long initial ablation time, immediate and delayed complication rates, VRR, initial ablation rate (IAR), number of RFA sessions required for a complete treatment, long-term nodular recurrence rate, and the association with factors influencing therapeutic success are issues warranting further exploration [13, 28–30]. Determining if predictive criteria of the safety and effectiveness of RFA exist, despite the fact that we forecast the group of patients with a high risk of requiring more treatment sessions, was of special interest in this study. We thus investigated the potential factors influencing the therapeutic success of RFA for huge BTNs (volume >100 ml) and evaluated the feasibility of RFA as an alternative treatment modality for patients with a high risk of anesthesia or concern of postoperative scar and lifetime thyroxine supplementation due to hypoparathyroidism.

## 2. Materials and Methods

### 2.1. Patients

From May 2017 to January 2022, a total of 868 patients underwent RFA for BTNs treatment at the Kaohsiung Chang Gung Memorial Hospital Medical Center in Taiwan, of which 29 patients presented with a BTN volume >100 ml (defined as huge BTNs). Patients with huge BTNs presented with cosmetic issues, nodule-related problems/symptoms, and sought treatment options aside from surgery. Patients had visited otolaryngologists, internal medicine physicians, or surgeons and were subsequently transferred to the Radiology Diagnostic Department for sonography to evaluate the thyroid nodular composition. As demonstrated earlier in the research by Kim and Lin et al. [3, 22], Ultrasound-guided core needle biopsy (CNB) or fine-needle aspiration cytology (FNAC) was performed for the benign nature of the nodules confirmation. At least a single benign cytological result or two benign cytological results with an acceptable ultrasound characteristic was considered at low risk of malignancy.

**Figure 1:**
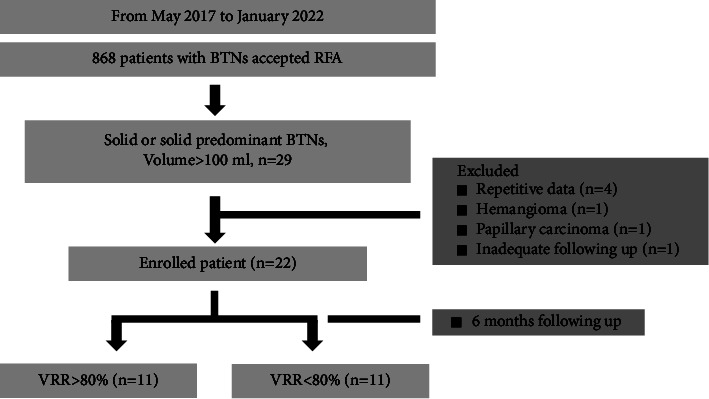
Consort diagram of the study.

In our study, all patients were without contraindications to surgery; however, these patients expressed concerns regarding postoperational complications, side effects, lifetime thyroxine supplementation due to thyroid functional changes, unpleasant scarring, or anesthesia risk. The demographic data for all patients were recorded, and their follow-up outcomes were analyzed retrospectively. The criteria for patient enrollment in the study were (1) age above 20 years, (2) symptomatic and/or cosmetic problems, (3) volume of thyroid nodule >100 ml, (4) solid or predominant solid/spongiform nodule, (5) cytological confirmation of benign nodule status by FNAC or CNB, (6) thyrotropin (TSH) and serum thyroid hormone (free T3 and free T4) levels within normal range, and (7) acceptance of RFA treatment. The exclusion criteria were patients with pathological results indicating malignancy or follicular neoplasm and patients without adequate follow-up sessions (at least 6 months). Excluded by exclusion criteria, 7 patients were excluded due to repetitive data (*n* = 4), pathology study revealed hemangioma (*n* = 1) and papillary carcinoma (*n* = 1), and inadequate follow-up time (*n* = 1). Finally, 22 patients presenting with huge BTNs who underwent RFA treatment were enrolled in the study (Figure 1). The retrospective study was approved by the Chang Gung Medical Foundation Institutional Review Board/IRB No. 202201363B0, which waived the obtaining informed consent requirement.

### 2.2. Preablation Status Evaluation and Preparation

At each visit to our Radiology Department, the thyroid function and nodule-related cosmetic score/symptom score were recorded. As shown previously in the study of Lin et al. [22] Patients will fill out a questionnaire which recorded as nodule-related symptom score focused on five clinical symptoms: cough, difficulty swallowing, compression, voice change, and pain. We allocated 1 point for each positive symptom; the symptom scores ranged from 0 to 5. The cosmetic score is obtained using the following scale: 0, no palpable or visible mass; 1, palpable mass but not visible; 2, only visible when swallowing; and 3, an easily visible mass [3]. In addition, sonography was used to evaluate the echogenicity of the thyroid nodule and measure the tumors' 3 orthogonal diameters (the largest diameter with two perpendicular diameters). CT/MRI will also be used to confirm these huge BTNs extend below the sternal notch or not and make sure the three orthogonal diameters of the tumors again. If not incompatible with both modality results, we will choose the largest one as the final result. Calculate the tumors' volume by using the following equation: *V* = *π* × *a* × *b* × *c*/6 (*V*: volume, *a*: the largest diameter, and *b* and *c*: the two perpendicular diameters)

### 2.3. Radiofrequency Ablation Procedure and Technique

All patients who accepted the RFA treatment are outpatient. The procedure was performed by an experienced radiologist with over 10 years of experience in the field of US-guided examinations and treatments. The patients were administered local anesthesia (solution of 2% lidocaine hydrochloride) at the puncture site and around the thyroid gland. According to examination guidelines of ultrasound, the tip size of electrode was chosen based on the status of the surrounding critical structures and tumor size. An internally cooled electrode (18 gauge, with 5/7/10 mm active tip) with RF generator (VIVA, STARmed and M2004, RF Medical) was applied, passing the electrode through the thyroid parenchyma by the transisthmic approach, with careful observation of the vessels along the approach route, into the thyroid nodule deepest portion. The moving shot technique was used sequentially for BTN ablation, which involves moving the electrode tip lateral to medial, bottom to top, and back and forth. When all visual fields of BTN had changed to transient hyperechoic zones, we terminated the ablation procedure. After the ablation, patients were referred to the Otolaryngology Department for examination by flexible fiberoptic laryngoscopy to check for occurrence of vocal cord paralysis.

### 2.4. Subsequent Post-RFA Follow-Up

**Figure 2:**
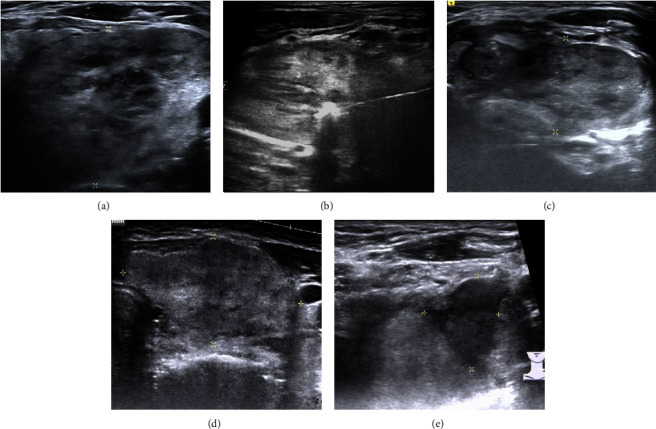
(a) Prior to RFA treatment, the 66-year-old with a heterogeneous echogenicity huge thyroid nodule on the left side of the neck region. (b) During the RFA treatment, the transisthmic approach was used by passing through the thyroid parenchyma, and the nodule was sequentially ablated using the moving shot technique until the visual fields of the nodule had changed to transient hyperechoic zones. (c) 1-month post-RFA treatment. (d) 3-month post-RFA treatment. (e) 6-month post-RFA treatment, revealing a distinct reduction in size.

**Figure 3:**
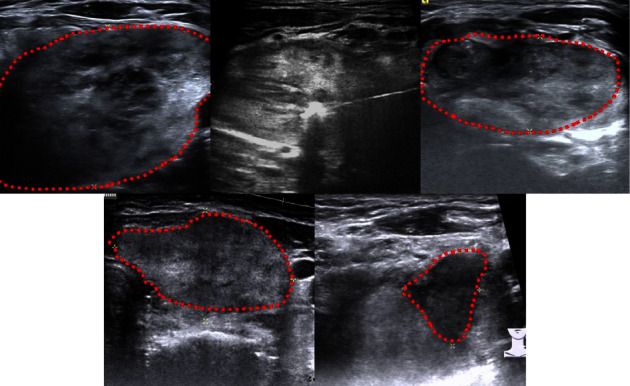
The red dots illustrate the nodule size from Figure 2.

Sonography was performed to assess the post-RFA VRR at 1, 3, and 6 months (Figures 2 and 3). The cosmetic score and symptomatic score were also documented at each follow-up. As shown previously in the study of Chen et al. [30], the measurement of IAR is based on the concept that the total nodule volume (*Vt*) can be divided into a vital portion (*Vv*) and an ablated (*Va*), i.e., *Vt* = *Vv* + *Va*. The IAR is calculated as follows: IAR = (*Va*/*Vt*) × 100 [30, 31]. The VRR is calculated as follows: VRR (y%) = initial volume (ml) − final volume (ml)  ×  100/initial volume, which is assessed by sonography. Every 6 months we will re-evaluate, if the patient still complained about the problem of symptom/cosmetic issues, subsequent RFA treatments may be needed and applied for residual tumor, and the post-RFA 6 months VRR was also recorded. Major and minor complications were evaluated according to the standard terminology of the Society of Interventional Radiology (SIR). Minor complications were defined as SIR classifications A-B: A: no therapy, nil consequence; B: nominal therapy, no consequence, included overnight admission for observation only. Major complications were defined as SIR classifications C-F: C: required therapy, minor hospitalization (<48 h); D: required major therapy, prolonged hospitalization (>48 h); E: permanent adverse sequelae; and F: death [32].

### 2.5. Statistical Analyses

SPSS, Version 22 (SPSS, Inc. Chicago, IL, USA), was used for results analyzation. We used two stages of statistical methodologies in this study. At the first stage, we analyzed the correlation of different factors between the two groups. Standard Chi-Square tests were used for group comparisons of categorical variables. Mann–Whitney *U* tests were used for group comparisons for continuous variables. The Friedman test for repeated measure analysis was used to assess relationship of VRR and nodule volume among the groups at each follow-up. The results are presented as odds ratios (ORs) with a 95% confidence interval (CI). The differences were considered significant when the *p* value was <0.05. If the data required two different statistical methods, the difference was considered significant when the results achieved *p* value <0.05 in each of the statistical methods. By including the total number of required RFA sessions and the favorable factors in the investigation, we can improve the predictive accuracy and enhance the treatment strategy. Thus, at the second stage, the significant factors identified at the first stage coupled with the total number of RFA sessions were entered into a stepwise multivariable logistic regression model to correlate the final post-RFA VRR at 6 months, with the statistical significance set at *p*  <  0.05.

## 3. Results

### 3.1. Demographic Characteristics and Influential Factors

All patients enrolled in the study received a single session of RFA treatment, while subsequent ablation sessions of the residual thyroid nodule were required if the patient had persistent cosmetic issues or symptoms. All BTNs were evaluated by sonography for confirmation of solid or predominant solid/spongiform nodule components. Demographic data of the 22 index huge BTNs based on the post-RFA VRR are presented in Table 1. Of the 22 huge BTNs, 11 nodules (50%) constituted the VRR >80% group, while the remaining 11 nodules (50%) constituted the VRR <80% group. In terms of the influential factors among the 22 patients, BTN characteristics including location on the left side, intrathoracic extension, predominant solid/spongiform BTNs, higher IAR, and lower resistance (watts) during the RFA procedure showed significant differences. Of note, there were no differences between the two groups in terms of sex distribution, age, pre-RFA BTN maximum length, original volume, total energy of the RFA procedure, energy per volume of RFA, total procedure time, original cosmetic scores, original symptomatic scores, the usage of electrode active tip, serum Ca, TSH, T3, or T4 levels.

### 3.2. Complications

The complications associated with the RFA treatment are presented in Table 2. A total of 3 (13.6%) immediate or delayed complications occurred after the RFA procedure, including 1 major complication and 2 minor complications [32]. The minor complications included 2 temporary vocal paralyses (2 patients: VRR >80% group, *n* = 1; VRR <80% group, *n* = 1). One patient with vocal paralysis (in VRR >80% group) recovered within 2 to 3 hours after the RFA procedure, while the other patient with vocal paralysis (in the VRR <80% group) recovered within 3 months after the RFA procedure. All of the minor complications belonged to SIR class B. One case of nodular rupture (in VRR >80% group), for which the patient was hospitalized for drainage (SIR class C), accounted for the major complication. The complication rate showed no significant difference among the 2 groups (3 patients: VRR >80% group, *n* = 2, %; VRR <80% group, *n* = 1, %, *p* = 1.00).

### 3.3. Odds Ratio

#### 3.3.1. Preprocedural Factors Influencing RFA

The factor of BTN location in the VRR >80% group increased the post-RFA 6-month follow-up VRR, with location on the left side at 81.8% vs. 18.2% on the right side (VRR <80% group: left side 36.4% vs. right side 63.6%) (OR 7.875; 95% CI 1.11–56.1, *p* = 0.03).

The factor of BTN echogenicity in the VRR >80% group increased the post-RFA 6-month follow-up VRR, with predominant solid/spongiform nodule 81.8% vs. 18.2% solid nodule (VRR <80% group: *p* predominant solid/spongiform nodule 36% vs. solid nodule 18.2%) (OR 7.875; 95% CI 1.11–56.1, *p* = 0.03).

#### 3.3.2. Periprocedural Factors Influencing RFA

The factor of total resistance (watts) during the RFA procedure in the VRR >80% group increased the post-RFA 6-month follow-up VRR, with lower resistance (watts) 45 (42.5, 54.5) vs. higher resistance (watts) 50 (45, 65) in the VRR <80% group (Mann–Whitney *u* test, *p* = 0.047). However, by applying logistic regression for odds ratio of continuous variables data, the statistical results revealed significant differences (OR 1.124; 95% CI 0.97–1.30, *p* = 0.121).

The overall median IAR was 63.3 (40.3, 71.1)%. A higher IAR in the VRR >80% group (70.6 (58.5, 73.5)%) was noted compared with VRR <80% group (51.4 (38.7, 64.5)%) with significant difference (*p* = 0.0028) (Table 1). The statistical results by logistic regression for odds ratio revealed OR 1.111; 95% CI 1.009–1.234, *p* = 0.01.

### 3.4. Volume and VRR

The BTN volumes of baseline and respective changes are presented in Table 3. Prior to the ablation procedure, the overall median BTN volume was 140.5 (114.6, 183.2) ml. After the ablation procedure, the 1-, 3-, and 6-month median BTN volumes were 59.9 (41.2, 68.1) ml, 44.6 (23.6, 57.0) ml, and 25.0 (17.2, 42.9) ml, respectively. The results showed that the overall nodular volume reduced significantly after RFA treatment over time (time effect, *p*  <  0.001). The volume in each VRR group also showed significant reductions over time (time effect, *p*  <  0.001). Although there was no significant difference (*p* = 0.797) between the baseline nodular volumes of the two groups, the volume at the 6-month follow-up showed a considerable difference between the 2 groups (group *∗* time effect, *p* = 0.052).

**Figure 4:**
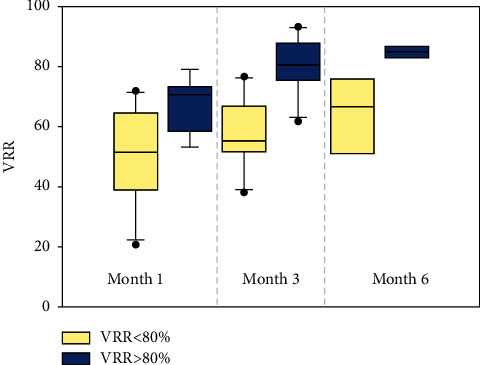
The 1-, 3-, and 6-month post-RFA treatment median VRR (%) of both groups. There was a statistically significant difference between both groups at 1-, 3-, and 6-month post-RFA treatment.

The 1-, 3-, and 6-month volume reduction rates are presented in Figure 4 and Table 3. The overall median volume reduction rates at the 1-, 3-, and 6-month follow-ups were 63.4 (40.4, 71.1)%, 73.1 (54.9, 81.1)%, and 82.4 (66.6, 85.3)%, respectively, revealing significant reductions over time (time effect, *p*  <  0.001). At the 1-, 3-, and 6-month follow-ups, the VRR showed a significant reduction in the VRR >80% group, with VRRs of 70.6 (58.5, 73.5)%, 80.5 (75.4, 87.7)%, 84.9 (83.0, 86.6)%, respectively, as compared to the VRR <80% group, with VRRs of 51.4 (38.7, 64.5)%, 55.2 (51.6, 66.9)%, and 66.6 (51.1, 75.8)%, respectively. The VRR of each group showed significant reductions over time (time effect, *p*  <  0.05); in addition, the VRR at the 1-, 3-, and 6-month follow-ups exhibited significant differences between the two groups (group ∗ time effect, *p* = 0.028, *p* = 0, and *p* = 0.004, respectively)

### 3.5. Symptom and Cosmetic Scores

Symptom and cosmetic scores are presented in Table 3. Prior to the RFA treatment, all patients (100%) had nodule-related symptoms, defined as a symptoms score >0. The overall symptom score improved from 2 (0, 2) to 0 (0, 0) at the 6-month follow-up (*p*  <  0.001), with each group achieving significant improvements (*p*  <  0.01). The symptom scores at baseline and the 6-month follow-up showed no significant difference between the two groups (*p* = 0.949 and *p* = 0.739, respectively). Prior to the RFA treatment, all patients (100%) had cosmetic concerns, defined as a cosmetic score >0. The overall cosmetic score improved from 3 (3, 3) to 2 (2, 3) at the 6-month follow-up (*p* = 0.001), with both groups achieving significant improvements (*p*  <  0.05). The cosmetic score at baseline and the 6-month follow-up showed no significant difference between the two groups (group *∗* time effect, *p* = 0.748 and *p* = 0.739, respectively).

### 3.6. Subsequent RFA Data and Multivariable Logistic Regression Analysis

The subsequent RFA data (total RFA time, IAR) and multivariable linear regression analysis are performed. In this study, a total of 22 patients underwent at least 1 RFA session; additionally, 6/22 patients underwent a total of 2 RFA sessions and 5/22 patients underwent a total of 3 RFA sessions. The median total number of RFA sessions was 1.5 (1.0, 2.25). 3 patients in VRR >80% group underwent a total of 2 RFA sessions, 3 patients in VRR <80% group underwent a total of 2 RFA sessions, and 5 patients underwent a total of 3 RFA sessions.

Three variables (BTN location, echogenicity (solid or predominant solid/spongiform nodule), and the IAR) showing statistical significance were coupled with the total number of RFA sessions and entered into a stepwise multivariable logistic regression analysis which revealed statistical significance (*F* = 3.852, *p* = 0.026). The analysis revealed that BTN echogenicity (standardized beta coefficients = 0.485, *p* = 0.047) and the IAR (standardized beta coefficients = 0.774, *p* = 0.004) were associated with the VRR. The regression analysis further revealed that BTN location (*p* = −0.25) and total number of RFA sessions (*p* = −0.112) were without significant difference. The regression model provides characteristics of normality (Shapiro–Wilk test *p* = 0.179), autocorrelation (Durbin–Watson = 2.594 which greater than dU = 1.543, alpha = 0.01 and dU = 1.797, alpha = 0.05) [33], and low collinearity (all variables' VIF <10 and condition index = 19.348).

## 4. Discussion

This retrospective study indicates that the factors influencing a favorable RFA treatment outcome of huge BTNs were more effective in the post-RFA 6-month VRR >80% group than those in the post-RFA 6-month VRR <80% group, while presenting an acceptable complication rate. Furthermore, the treatment effectiveness of RFA for huge BTNs was notable, with a mean VRR of 82.4 (66.6, 85.3)% at the 6-month follow-up. In addition, we reveal that huge BTNs characteristics including BTNs located on the left side (*p* = 0.03), predominant solid/spongiform BTNs (*p* = 0.03), and higher IAR (*p* = 0.028) are factors influencing a favorable VRR. The median RFA sessions count was 1.5 (1.0, 2.25). Additionally, the regression analysis model revealed that while predominant solid/spongiform BTNs and a higher IAR are associated with a superior VRR in patients with huge BTNs, the IAR is indeed a more influential factor than predominant solid/spongiform echogenicity. These findings may help clinicians to better educate and manage patients' expectations prior to RFA treatment and offer patients concerned about postoperational scarring or the risk of anesthesia a safe treatment option to preserve the thyroid.

Compared to 6-month post-RFA VRR, symptom, and cosmetic outcomes, the immediate short-term effects on huge BTNs after treatment are not inferior to average size BTNs. Previous studies have reported a VRR range of 52.1% to 86.1% at 6 months postablation for average-sized BTNs [34]. We similarly recorded a median VRR of 82.4% for huge BTNs at the 6-month follow-up. Because of the difficulty in achieving high IAR and higher rates of regrowth, the patients in this study received 1.5 RFA treatments on average with further treatments. However, future treatments are usually needed [14, 18, 19, 35]. In comparison to a previous study of large BTNs (defined as >30 ml, *n* = 44, 6-month post-RFA symptom, and cosmetic score: 0.05 ± 0.2 and 1.3 ± 1.0, respectively), our symptom score at 6 months post-RFA exhibits superior improvement for the huge BTNs [22]. In several other papers on large nodules, though not as large as in this study, the baseline symptom score was much higher; the nodules in these studies reduced after treatment but not to such low symptom score values [22, 36, 37]. While this may arise from the fact that symptom and cosmetic scores are subjective factors, the tremendous shrinkage of the huge BTNs may account for great remission feedback among patients. Additionally, the patients reported decreased self-consciousness about their thyroid issues. Meanwhile, our results also indicate that subsequent RFA procedures may be necessary to achieve complete resolution of cosmetic issues, despite greater cosmetic score improvement at 6 months post-RFA than in previous studies [5, 22]. This comparison is not appropriate, however, with studies where the volume reduction was equally significant but where the nodules were subject to a single treatment. Although patients with huge BTNs can receive thyroidectomy, RFA is not always the first priority treatment due to its high ratio of regrowth as well as cost and quality of life concerns.

The IAR was revealed to be a major factor influencing the VRR of huge BTNs in our study. The VRR >80% group exhibited a better IAR than the VRR <80% group, demonstrating that the IAR is a quantitative indicator of the performance efficacy of the RFA procedure and is highly correlated with the VRR [30, 38]. Some studies suggest that smaller or medium-sized nodules have better VRR than large-sized BTNs in long-term effects because of better IAR [18, 19, 35]. Chen et al. reported no difference in terms of nodule volume and achieved an average IAR of 99.67% [30], although their study did not include such huge BTNs as are investigated here. Indeed, it is challenging to reach a 70% IAR for huge BTNs due to issues related to tumor size, device limitations, and technical factors [39]. The nodules in our study have undergone more than one retreatment over 6 months (1.5 treatments on average). This likely results from the nodules not reaching a sufficiently high IAR, in line with previous work that found more treatments are needed for larger-size BTNs [14]. One particular issue to achieving a high IAR lies with the residual margin [30]. Although leaving a relatively large margin is safe from the perspective of complications, leaving too much margin may lead to therapeutic failure [38]. It is important to treat the margin completely in order to prevent the regrowth of marginal viable tissue around the central ablated tissue [30, 40]. Hence, subjecting the margin of huge BTNs to additional treatment is critical, which may require the patient to accept additional sessions of RFA [14]. Considering the higher regrowth rate among these patients, for whom repeat treatment is likely, compared to those who were treated only once (for smaller BTNs) or surgically operated on, RFA can only be an alternative treatment [35, 41].

Another factor identified in our study influencing a favorable treatment outcome was echogenicity. Previous studies have reported that <30 ml mixed cystic BTNs showed a significantly better volume reduction response than predominant solid/spongiform BTNs after RFA treatment [4, 34]. Specifically, one study reported a 6 to 9 month post-RFA residual BTN volume of 21.9% ± 16.5% in a mixed cystic group and 50.0% ± 31.4% in a predominant solid/spongiform group [34]. This may be attributed to the homogeneous conduction of heat and the absence of a heat sink effect [42]. Given that cystic content, necrosis, and hemorrhage can produce heterogeneous echogenicity [43], predominant solid/spongiform echogenicity in the BTNs studied here (>100 ml) may also have superior heat conduction and a less limiting heat sink effect. On a procedural note, as the predominant solid/spongiform echogenicity exhibited by huge BTNs can provide a clearer RFA target and BTN border, the operator may have the opportunity to ablate the huge BTNs in a more detailed manner.

We also observe that huge BTNs located on the left side have better VRR than those on the right after odds ratio analysis. We presume the relative ease of the operator using their dominant hand to manipulate the RFA needle while performing treatment at the patient's cephalic site; thus, as the operator in this study was right-handed, they could handle the sonography probe with the left hand and the RFA needle with the right hand. In this way, the operator could ablate the huge BTNs in a detailed manner to treat the margin more completely, which could lead to improved IAR. However, further regression analysis did not reveal this factor to be significant. The reduced number of nodules enrolled in the two groups and operator-dependent factors should also be considered.

The energy delivered per nodular volume factor showed no statistical difference between the groups of huge BTNs. Previous studies have suggested that the energy delivered per volume was independently predictive of volume reduction [22, 39, 44]. Deandrea et al. reported energy superior to 2670 J/ml could facilitate an optimized treatment efficacy, with a VRR >50% in 99% of cases [44]. Furthermore, studies have reported the best volumetric response to RFA in small (<15 ml) and medium (15–30 ml) nodules, where the energy delivered was higher [22, 44]; nevertheless, such a correlation has not been found in large BTNs [22]. Considering huge BTNs present a more complicated nodular pattern, such as intense vascularity, microcystic composition, and soft stiffness, and further study is warranted to investigate the correlation.

The complication rate associated with RFA treatment of huge BTNs in this study was acceptable, in line with findings of previous studies and without incidence of life-threatening complications [45]. Indeed, surgery remains the gold standard for treatment of large-size thyroid nodules or those presenting with compressive symptoms or nodular growth, as it can completely remedy the compressive symptoms caused by nodular volume [25, 46, 47]. It must be noted that several studies have revealed common complications specific to thyroidectomy: temporary vocal paralysis, which occurs in 5% to 11% of cases and may be permanent in 1% to 3.5% of cases; temporary hypoparathyroidism, which occurs in 20% to 30% of cases and may be permanent in 1% to 4% of cases [48–50]; and postoperative hematoma, which occurs in 1.9% to 14.3% of cases [51]. No patient in this study had hypoparathyroidism, postoperative hematoma, or other permanent complications, with a complication rate not higher than that of thyroidectomy. More specifically, 2 patients (9.1%) had temporary vocal paralysis, accepted immediate steroid IV injection, and were transferred to the ENT Department for follow-up, recovering within 2 hours and 3 months, respectively, without further hospitalization. Potential mechanisms associated with vocal cord paralysis include nerve stretching during the RFA procedure, hemorrhage [52], lidocaine injection, and RFA-induced thermal injury rather than permanent nerve damage [45, 53–56]. In addition, 1 patient (4.5%) suffered a nodular rupture, presenting with redness of the neck region accompanied with neck pain 2 weeks following the RFA treatment. After receiving debridement and antibiotic treatment, the patient recovered within 3 weeks. Post-RFA BTN rupture may result from tearing of the tumor wall and thyroid capsule at a weak point [29, 39, 45, 56–58]. Other potential causes of nodule rupture include large nodular size, location near the anterior thyroid capsule, solid component, excessive RFA power, and longer ablation time [22]. In RFA treatment of BTNs, life-threatening complications including injury to the trachea and esophageal rupture have not been reported [59]. Taken together, this study reveals that the complication rate associated with RFA treatment of huge BTNs is not higher than that of surgery, while further investigation is necessary to evaluate the correlation between nodular size and the aforementioned complications. Besides, a recent study demonstrated a small risk of malignancy in thyroid nodules >4 cm despite benign FNA results [60]. Following the 2021 Asian and 2020 European guidelines of RFA in BTNs, at least two FNAC or core needle biopsy (CNB) was performed to confirm the nodule's benign nature and informed the patients of the risk of malignancy [1, 5]. In our study, the patients were unwilling to have surgery and fully understood the small potential malignant result. All in all, the surgery is the gold standard for this group of patients, and RFA is a modality treatment for patients unable or unwilling to the surgery.

This study has several limitations. First, as a retrospective single-center study, uncontrolled bias could have been introduced. Second, this study included a small group of patients and a relatively short-term following-up period. More patients and further long-term analysis are needed. Third, the treatment aims are different from usual-sized nodules and other thermoablation techniques, and there are more than 50% of them who were subjected to several treatment sessions. It is hard to compare the effect with previous studies. Fourth, as RFA is an operator-dependent treatment, hardware, software, and experience may influence the ablation results. Fifth, huge BTNs exhibit more variable and ill-defined nodular margins, and thus nodule location close to the danger triangle area or carotid artery, prominent peripheral vascularization [44], macrocalcifications, periprocedural tissue temperature [58], and peripheral, internal vascularity are all factors potentially influencing VRR differences. Further prospective studies focused on elucidating these factors are recommended.

## 5. Conclusions

This study demonstrates that RFA is an effective alternative treatment modality with an acceptable complication rate for patients presenting with huge BTNs who are unable or unwilling to undergo surgery. Critically, RFA treatment of huge BTNs presents unique challenges to patients and physicians, wherein incomplete resolution or relapse of symptomatic or cosmetic issues may occur, and further treatment sessions are usually needed. It is thus recommended that physicians discuss these risks with patients during the treatment decision-making process.

## Figures and Tables

**Table 1:** The 22 possible influence factors of huge BTNs based on the post-RFA VRR.

Characteristics	VRR <80%	VRR >80%	*p* value
Gender			0.611
Male	3 (27.3%)	2 (18.2%)	
Female	8 (72.7%)	9 (81.8%)	
Age (year)			1
<50 y/o	5 (45.5%)	5 (45.5%)	
>50 y/o	6 (54.5%)	6 (54.5%)	
Location			0.03^*∗*^
Left	4 (36.4%)	9 (81.8%)	
Right	7 (63.6%)	2 (18.2%)	
Intrathoracic extension			0.665
With	5 (45.5%)	4 (36.4%)	
Without	6 (54.5%)	7 (63.6%)	
Echo feature I			0.03^*∗*^
Predominant solid/spongiform	4 (36.4%)	9 (81.8%)	
Solid	7 (63.6%)	2 (18.2%)	
Echo feature II			1
Hyperechoic	9 (81.8%)	9 (81.8%)	
Hypoechoic	2 (18.2%)	2 (18.2%)	
Electrode active tip			1
10 or 15 mm	11 (100%)	10 (90.9%)	
5 mm	0 (0%)	1 (9.1%)	

Characteristics	VRR <80%^a^	VRR >80%^a^	*p* value

Max length (cm)	8.8 (8, 10, 8)	8.9 (7.9, 10.1)	0.797
Original size (mL)	128.3 (108.1, 212.8)	141.8 (124.5, 160.8)	0.797
Total RFA energy (J)	28.7 (23.9, 47.6)	32.2 (17.7, 43.9)	0.478
Energy/volume (J/mL)	0.2 (0.1, 0.3)	0.2 (0.1, 0.3)	0.438
Initial ablation rate (%)^b^	51.4 (38.7, 64.5)	70.6 (58.5, 73.5)	0.028^*∗*^
Mean resistance (watts)	50 (45, 65)	45 (42.5, 54.5)	0.047^*∗*^
Total RFA time (min)	40 (35, 55)	46 (27.5, 72)	0.748
Original symptom score^c^	2 (1, 2)	2 (0, 2)	0.949
Original cosmetic score^d^	3 (3, 3)	3 (3, 3)	0.748
Original iPTH (pg/mL)^e^	28.5 (14.8, 45.1)	49.8 (19.1, 80.1)	0.195
Original Ca (mg/dL)	9.4 (9.0, 9.9)	9.4 (9.1, 9.9)	0.888
Original T3 (ng/dL)	98.2 (80.6, 115.6)	100 (87.6, 111.2)	0.973
Original T4 (ng/dL)	1.2 (1.0, 1.3)	1.2 (1.0, 1.4)	0.918
Original TSH (*μ*IU/mL)	1.0 (0.4, 1.2)	0.6 (0.1, 1.0)	0.197

^*∗*^Statistically significant difference. ^a^Data are presented as median (25^th^ percentile and 75^th^ percentile). ^b^Initial ablation rate (%) = (ablation region volume/total volume) × 100. ^c^For each positive symptom, we allocated one point; therefore, the symptom scores ranged from 0 to 5. ^d^The cosmetic score was obtained using the following scale: 0, no visible or palpable mass; 1, not visible but palpable mass; 2, visible when swallowing only; 3, an easily visible mass [3]. ^e^Intact parathyroid hormone.

**Table 2:** Post-RFA complications for two groups.

Complications	Total	VRR <80%	VRR >80%
Vocal palsy	2	1	1
Burn	0	0	0
Hematoma	0	0	0
Rupture	1	0	1
Total	3	1	2

**Table 3:** The 1-, 3-, and 6-month follow-up median volume and volume reduction ratio (%) and median symptoms and cosmetic score at baseline and at 6 months post-RFA treatment.

	Total^*∗*^	VRR <80%^*∗*^	VRR >80%^*∗*^	Group *∗* time effect *p* value
*Nodule volume*				
Baseline	140.5 (114.6, 183.2)	128.3 (108.1, 212.8)	141.8 (124.5, 160.8)	0.797^a^
1 month	59.9 (41.2, 68.1)	65.7 (59.9, 76.1)	41.2 (32.0, 58.3)	
3 months	44.6 (23.6, 57.0)	48.8 (45, 62.1)	24.2 (17.4, 35.9)	
6 months	25.0 (17.2, 42.9)	42.9 (24.6, 59.3)	19.5 (15.3, 28.5)	0.052^b^
Time effect *p* value	<0.001^c^	<0.001^d^	<0.001^e^	(Friedman test)

*VRR*				
1 month	63.4 (40.4, 71.1)	51.4 (38.7, 64.5)	70.6 (58.5, 73.5)	0.028^f^
3 months	73.1 (54.9, 81.1)	55.2 (51.6, 66.9)	80.5 (75.4, 87.7)	0^g^
6 months	82.4 (66.6, 85.3)	66.6 (51.1, 75.8)	84.9 (83.0, 86.6)	0.004^h^
Time effect *p* value	<0.001^i^	<0.05^j^	<0.05^k^	

*Symptoms score*				
Baseline	2 (0, 2)	2 (1, 2)	2 (0, 2)	0.949^l^
6 months	0 (0, 0)	0 (0, 0)	0 (0, 0)	0.739^m^
Time effect *p* value	<0.001^n^	0.005^o^	0.008^p^	

*Cosmetic score*				
Baseline	3 (3, 3)	3 (3, 3)	3 (3, 3)	0.748^q^
6 months	2 (2, 3)	2.5 (2, 3)	2 (2, 3)	0.739^r^
Time effect *p* value	0.001^s^	0.025^t^	0.014^u^	

^*∗*^Data are presented as median (25th percentile and 75th percentile). ^a,b^There was no significant difference in the volume of both groups at baseline and at 6 months post-RFA treatment. ^c,d,e^Volume and time effect of both groups showed significant difference. Comparison of both groups for time effect showed significant difference. ^f,g,h^There was a significant difference in the VRR of both groups at the 1-, 3-, and 6-month post-RFA follow-ups. ^i,j,k^VRR and time effect of both groups showed significant difference. Comparison of both groups for time effect showed significant difference. ^l,m^There was no significant difference in the symptom scores of both groups at baseline and at 6 months post-RFA treatment. ^n,o,p^Symptom scores and time effect of both groups showed significant difference. Comparison of both groups for time effect showed significant difference. ^q,r^There was no significant difference in the cosmetic scores of both groups at baseline and at 6 months post-RFA treatment. ^s,t,u^Cosmetic scores and time effect of both groups showed significant difference. Comparison of both groups for time effect showed significant difference.

## Data Availability

The data used to support the findings of this study were supplied by professor Wei-Che Lin under license and so cannot be made freely available. Requests for access to these data should be made to Chun-Hua, Chiu, email: photododophotododo@hotmail.com.
